# Micro-Engineered Models of Development Using Induced Pluripotent Stem Cells

**DOI:** 10.3389/fbioe.2019.00357

**Published:** 2019-11-29

**Authors:** Pallavi Srivastava, Kristopher A. Kilian

**Affiliations:** ^1^School of Medical Sciences, University of New South Wales, Sydney, NSW, Australia; ^2^Australian Centre for Nanomedicine, School of Chemistry, School of Materials Science and Engineering, University of New South Wales, Sydney, NSW, Australia

**Keywords:** development, gastrulation models, micropatterning, biomaterials, iPS cells, morphogenesis

## Abstract

During fetal development, embryonic cells are coaxed through a series of lineage choices which lead to the formation of the three germ layers and subsequently to all the cell types that are required to form an adult human body. Landmark cell fate decisions leading to symmetry breaking, establishment of the primitive streak and first tri-lineage differentiation happen after implantation, and therefore have been attributed to be a function of the embryo's spatiotemporal 3D environment. These mechanical and geometric cues induce a cascade of signaling pathways leading to cell differentiation and orientation. Due to the physiological, ethical, and legal limitations of accessing an intact human embryo for functional studies, multiple *in-vitro* models have been developed to try and recapitulate the key milestones of mammalian embryogenesis using mouse embryos, or mouse and human embryonic stem cells. More recently, the development of induced pluripotent stem cells represents a cell source which is being explored to prepare a developmental model, owing to their genetic and functional similarities to embryonic stem cells. Here we review the use of micro-engineered cell culture materials as platforms to define the physical and geometric contributions during the cell fate defining process and to study the underlying pathways. This information has applications in various biomedical contexts including tissue engineering, stem cell therapy, and organoid cultures for disease modeling.

## Introduction

The physical microenvironment of a cell population *in-vivo* has been demonstrated to play a central role in collective cell behavior, fate-determination and spatio-temporal orientation (Gattazzo et al., 2014; Ahmed and Ffrench-Constant, 2016). In conjunction with cytokine signaling, the crosstalk between the extracellular matrix and cells create a state of “dynamic reciprocity” which guides the form and function of a living organism (Bissell et al., 1982; Lu et al., 2011). This dynamic reciprocity is a function of the biophysical and biochemical aspects of specific niches during development and sets a context in which these signals are integrated to regulate gene expression programs. Dynamic changes in the microenvironment underlie all morphogenetic processes leading to a need for laboratory models to study development and disease. However, recreating the complex interplay between the matrix and cells is challenging using conventional cell culture materials. Hydrogel-based biomaterials that better reflect the physical and chemical properties of tissue have been deployed to evaluate adult stem cell lineage determination, including the role of matrix viscoelasticity (Discher et al., 2005; Evans et al., 2009; Chaudhuri et al., 2015; Das et al., 2015) and geometry (Kilian et al., 2010; Higuchi et al., 2013; Lee et al., 2013; Werner et al., 2017). In attempts to closely mimic the *in-vivo* microenvironment, hydrogels, microcarriers, scaffolds and other biomaterials have been used to drive the differentiation of pluripotent stem cells (PSCs) into either embryoid bodies (EBs—aggregates of PSCs exhibiting multilineage gene expression) or more specific cell lineages, as reviewed in detail by Higuchi et al. (2017). These microenvironment parameters have been shown to promote physiologically relevant bioactivities in cells compared to when grown on a hard-polystyrene surface of a tissue culture plastic dish. Assessing cell response to a combination of these parameters in 3D would most closely reflect the *in-vivo* environment of a complex system like a gastrulating human embryo and is elemental for a systems level understanding of the cell-lineage determination process. However, creating a platform with spatiotemporal control of microenvironment cues to study the dynamic signaling during embryogenesis remains a challenge.

**Figure 1:**
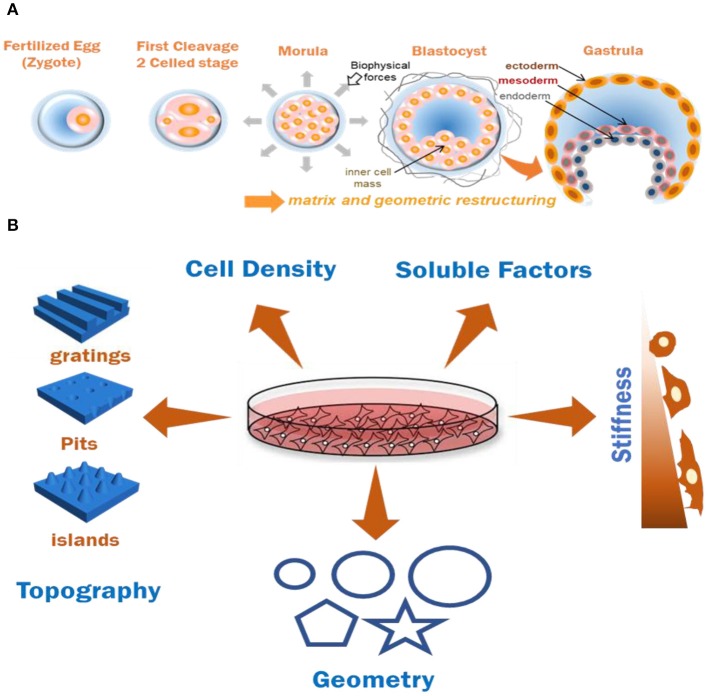
**(A)** Graphical representation of effects of biomechanics and geometry in coordinating cell organization and germ-layer differentiation during embryonic development. **(B)** Various biochemical and biomechanical factors which have been documented to induce stem cell (MSC and PSCs) differentiation patterns *in-vitro*. Combining two or more of these factors for documenting cumulative cell responses is useful for understanding ECM based tissue modeling approaches.

The process of human gastrulation is coordinated by the cumulative effects of the biophysical and biochemical environment with tight coordination of multivariate cues underlying cell-fate determination (Figure 1A). A thorough illustration of the process remains elusive due to the limitations of studying a live human embryo. Various groups have tried recapitulating the gastrulation process *in-vitro* using the self-organization potential of PSCs, including embryonic stem (ES) cells, epiblast-like cells (EpiLC), and induced pluripotent stem cells (iPS cells) (Warmflash et al., 2014; Deglincerti et al., 2016b; Shao et al., 2017). Such studies simplify the complexities of *in-vivo* tissue, by untwining the effects of individual stimuli toward enabling the researcher to ask directed questions related to developmental processes. In this review article, we describe the physical microenvironment in the development of the implanted embryo, and then delve into how laboratory models based on micro-engineered cell culture platforms control mechanics and topography to guide stem cell differentiation. Finally, we critically evaluate the current status of developmental models using PSCs and discuss how biomechanical manipulation can be deployed for an *in-vitro* gastrulation model using iPS cells. Considering the wealth of information gathered over the past two decades using adult stem cell systems [e.g., mesenchymal stem cells (MSCs)] and embryonic stem cells (ESCs) in bioengineering research, each section will give examples from these fields to set the stage for current and future work using micro-engineered models from iPSCs.

## Developmental Contributions of Biomechanical Factors in Uterine Development

The implantation of an embryo onto the maternal endometrial tissue is a major event towards a successful early mammalian development and involves high levels of maternal investment as opposed to development in other classes of organisms. As reviewed by McGowen et al. (2014), an interstitial embedding of the blastocyst and complete encasement of the embryo by endometrial tissue before tri-lineage differentiation is observed in only four orders in the class of mammalians—Rodentia (rodents), Chiroptera (bats), Eulipotyphla (moles and hedgehogs), and Primates (apes and humans). These surrounding tissues control the architectural and geometric environment of the embryo, and the developmental functions of these biophysical cues have been characterized in mice as being a representative species but remain largely unexplored in their human counterpart.

The mechanical and geometric constraints experienced by an implanted gastrulating embryo are known to have crucial roles in developmental functions (Figure 1A). Since the axis determination stage has been recapitulated *in-vitro* and away from uterine wall constraints, it was believed that embryonic axes specification is a uterus-independent process (Rossant and Tam, 2004). However, *ex-vivo* experiments which involved growing embryos in soft poly-dimethylsiloxane (PDMS) or agarose channels of varying diameter were performed to assess the effects of the applied microenvironment forces. It was shown that the exerted physical strains caused localized breach of basement membrane and transmigration of a population of cells poised to become Distal Visceral Endoderm (DVE) (Hiramatsu et al., 2013). Physical stresses and anisotropic forces from extra-embryonic and embryonic tissues and surrounding fluids on the EPI cells were documented to drive the convergent-extension of cells during notochord morphogenesis (Imuta et al., 2014). Moreover, as the function of transmembrane integrins (Darribère et al., 2000) being the major class of receptors assisting cellular response as well as of metalloproteases like ADAM10 (Alfandari et al., 2009) have been widely accepted in cellular functions and embryogenesis, the co-relation of their function with the effects of local physical forces is undeniable. Apart from mechanical forces from uterine and extra-embryonic tissues, there are frictional and tensional forces (Freund et al., 2012), and hydrostatic pressures (Navis and Bagnat, 2015) experienced by the embryo. These fluids convey crucial morphogenic information, yet their impact on development is still under exploration (Smith et al., 2019). Along with the mechanics, the spatial positioning of the embryonic cells guided by the geometry of the local niche plays a crucial role in organizing the BMP4 cell signaling gradient in the embryo owing to the localization of the receptors as well as the organization of extra-cellular spaces for ligand presentation (Zhang et al., 2018). The self-organization and T/Brachyury (T/Bra) cell localization in mES cells has been shown to be a function of the colony shape and curvature (Blin et al., 2018). In case of sea anemones, embryo compression resulted in ectopic expression of gastrulation marker *T/Bra*, identifying a key gastrulation gene whose expression could be induced by mechanical forces, and suggesting an evolutionarily conserved contribution of biomechanical forces toward embryonic development (Pukhlyakova et al., 2018). Collectively, these results demonstrate an indispensable role of environmental geometry and mechanical cues, in the axes formation and in directing differential responses in embryonic cells. Observing the individual effects of these cues could help disentangling the indirect or direct effects of each of these biomechanical forces in the differentiation of embryonic cells.

Manipulation of embryos *in-vitro*, and their response to various physical stimuli would prove useful in understanding this process. However, ethical concerns have prohibited the culture and research of human embryos beyond 14 days of development (Pera, 2017). This is approximately the time of appearance of the primitive streak (PS) in the developing human embryo. Although morphological data for each developmental stage has been collected, the cell signaling dynamics underlying the cell fate changes has little to no reports in humans. Lately, a few groups reported the polarization and self-organization of human embryos from *in-vitro* fertilization (IVF) processes when cultured *in-vitro*, but many aspects like amnion fate determination or primitive streak formation are unexplored due to ethical limitations and complexity of the interconnectedness of the developmental processes (Deglincerti et al., 2016a; Shahbazi et al., 2016).

Recent advances in developmental biology, which suggest the possibility of formation of functioning embryoid bodies through 3D synthetic approaches, have led to scientific and ethical discussions toward revisiting and extending the 14 day rule to a 28 day rule, to advance scientific understanding of human development during this period (Hurlbut et al., 2017; Appleby and Bredenoord, 2018; Chan, 2018). While most of the relevant current information on the subject heavily relies on the research on mouse embryos, the only possible means of research on changes occurring in post-implantation human embryo rely on *in-vitro* 2D or 3D approaches using pluripotent stem cells, or by comparison with non-human primate embryos. Therefore, a reliable and reproducible *in-vitro* platform to study gastrulation and underlying mechanisms for lineage differentiation using easily accessible human iPS cells would prove to be useful considering the limitation to directly study an intact developing human embryo. These limitations call for alternative platforms which can be representative of the *in-vivo* microenvironment, towards deconstructing the interconnected morphogenetic pathways during the process of development.

## ECM Guided Differentiation of Stem Cells: Significance of Mechanics and Geometry

Tissue assembly is largely based on the migration, adhesion and orientation of the cells, processes which are assisted by focal adhesion structures (Geiger et al., 2009) and the interaction between adaptor proteins, receptors, and the cytoskeleton. This machinery which assists in biomechanical sensing plays major roles in all cellular functions as well as disease progression, as reviewed by Vogel and Sheetz, where they described the phenomenon by which cells respond to biomechanical cues (Vogel and Sheetz, 2006). Firstly, the cell's initial response after sensing the topological cues by initiating a molecular change, termed mechanosensing. Secondly, translating these molecular changes toward creating a biochemical signaling response, termed mechanotransduction. And lastly, the overall changes of the cellular and molecular dynamics of the cell toward the newly created microenvironment, termed mechanoresponses. Hence, understanding this dynamic crosstalk between cells and their environment, and the development of laboratory models that can assist this process, is a critical need for deconstructing morphogenesis.

### Matrix Mechanics—An Overarching Physical Cue for Guiding Lineage Specification

The stiffness of the substratum in which a cell attaches plays a decisive role in morphology and behavior. Young's modulus or elasticity is the ability of a material to resist deformation in response to an external force. The tissue culture polystyrene (TCP) plates traditionally used to attach cells in stem cell biology have a substrate stiffness on the order of ~3 gigapascals (GPa) (Eyckmans and Chen, 2014). The plastic culture surfaces lack spatial domains and are flat and rigid (Walters and Gentleman, 2015). For more sensitive cell populations, TCP is often coated with animal derived ECM- protein(s) (e.g., collagen, fibronectin, matrigel, etc.) or feeder cell layers to better mediate cell attachment and proliferation. While this has led to identification of material combinations to support the viability and long-term culture of stem cells (Chen et al., 2012; Gattazzo et al., 2014), these materials do not reflect the native mechanics of embryonic tissue. Therefore, considerable efforts have been made to recreate and integrate the diverse set of natural biomechanical and biochemical cues to observe how they induce a specific response in cells, as opposed to when they are cultured on polystyrene surfaces (Evans et al., 2009; Smith and Gerecht, 2016; Vining and Mooney, 2017).

As described by Tsou et al., various combinations of natural (hyalurinic acid HA, alginate, chitosan, collagen, gelatin, etc.) and synthetic [polyacrylamide(PA), poly-ethylene glycol, poly vinyl alcohol, etc.] hydrogels have been tested in 2 and 3 dimensional cell culture (Tsou et al., 2016; Choe et al., 2018) owing to their biodegradability and biocompatibility and the results clearly indicate a crucial role of substrate stiffness in cell proliferation, migration, adhesion and differentiation (Higuchi et al., 2013; Figure 1B). The concentration of a biopolymer and crosslinking density of the hydrogel can be tuned to vary the mechanics of a substrate. Natural polymers can mimic native ECM to an extent and are prone to enzymatic degradation by cells, a process which can be tuned by altering the stage of secondary crosslinking, resulting in altered subsequent differentiation behavior of the cells (Khetan et al., 2013). Synthetic hydrogels like PA are generally inert toward cell adhesion, hence allowing the researcher to define and control their preferred adhesion ligand type and their presentation patterns through various physical or chemical means (Lee et al., 2013). The stiffness of synthetic hydrogels is tunable, and hence can be used to create soft (<1 kPa), moderately stiff (10–15 kPA), and rigid (>30 kPa) materials and be used to alter the differentiation pattern of stem cells. This control over elasticity has been shown to induce neuron-like cells from MSCs when cultured on soft polyacrylamide hydrogel substrates, myoblast-like cells when cultured on stiffer substrates, and osteoblasts when cultured on relatively rigid substrates, underlining the importance of mechanics in guiding cellular decision making (Engler et al., 2006; Lanniel et al., 2011; Haugh et al., 2018; Hiew et al., 2018; Sun et al., 2018; Gerardo et al., 2019).

Cells respond to the elasticity of the substrate via intergrin-mediated focal adhesion signaling. Integrins family of receptors comprise of α and β chain transmembrane heterodimers which mediate the cell-matrix crosstalk by activating important biochemical signals (for instance, phosphatase and tyrosine kinase) for the regulation of stem cell fate (Chowdhury et al., 2009). Moreover, increased activities of Rho kinase (ROCK), ERK1/2 and focal adhesion kinase (FAK) were noted to induce osteogenic differentiation in MSCs cultured on PA hydrogels, highlighting their crucial role as mechanotransducers (Shih et al., 2011). This substrate stiffness-based differentiation of stem cells was also deployed to identify specific lipid metabolites like lysophosphatidic acid and cholesterol sulfate, regularly being depleted during chondrogenesis and osteogenesis, respectively (Alakpa et al., 2016). Rather than these bioactivities being related to bulk modulus alone, several groups have demonstrated how other mechanical properties play key roles in propagating downstream cell signaling including stress relaxation and stiffening of natural materials (Chaudhuri et al., 2015; Das et al., 2015) and how matrix mechanics influences the presentation of integrin ligand in response to cell generated forces (Kilian and Mrksich, 2012; Trappmann et al., 2012; Wen et al., 2014).

The role of substrate stiffness as a contributor to the maintenance of pluripotency in ESCs has been studied (Blin et al., 2010) as well as softer substrates promoting Mesenchymal-to-Epithelial (MET) transition during the cellular reprogramming of mouse fibroblasts to iPS cells (Choi et al., 2016). Stiffness influences germ layer commitment in ESCs (Zoldan et al., 2011), where softer substrates promote higher expression of endoderm related genes (Sox17, AFP) in both 2D and 3D culture conditions (Jaramillo et al., 2015), and stiffer substrates promote mesodermal gene expression (Brachyury) (Evans et al., 2009; Dado-Rosenfeld et al., 2015). More recently, Maldonado and colleagues reported the development of a semi-3D and spherical arrangement of an iPS cell colony on a softer electrospun nanofiber substrate, as compared to a 2D cell colony on a stiffer substrate (as illustrated in Figure 1B), primarily affected iPS cells during lineage specific differentiation (Maldonado et al., 2016, 2017). They reported enhanced efficiency of biochemically induced mesoendodermal differentiation on stiffer substrates (based on the expression of mesoendodermal lineage markers MIXL1 and Brachyury) as compared to an embryoid body (EB) based differentiation method. Whereas, the semi-3D and semi-spherical iPS colony on softer substrate exhibited enhanced ectodermal differentiation (based on lineage markers-PAX6 and NEUROD1). While comparing the behavior of human iPS cells on surfaces containing elastomeric PDMS pillars of varying modulus, Chen and colleagues reported that intermediate elasticity of 9 kPA was the most suitable for EB body aggregation and cardiomyocyte differentiation (Wang et al., 2018). Collectively, such results support the idea that matrix mechanics exerts an influence on embryonic development (Choi et al., 2016), homeostasis (tissue maintenance) (Gattazzo et al., 2014) and disease processes (Cancerous tumor progression) (Northcott et al., 2018) thereby necessitating *in-vitro* cell culture models where rigidity can be tuned appropriately.

### Matrix Properties Spatially Organize Cells in Defined Geometries

Key developmental milestones during interstitial embryonic development have been linked to biomechanics of the confined embryo (Hiramatsu et al., 2013); its geometric architecture has been reported to be directly linked to the regulation of early signaling gradients (Zhang et al., 2018). A stem cell's mechanosensing of the nanoscale features and orientation of ligand presentation through their matrix is fundamental toward various cellular responses *in-vivo* and has been experimentally validated in 2D systems. Changes in cytoskeletal structure and tension have been postulated to be the guiding force behind the alternating behavior of cells when presented with various geometric cues. Enhanced actin stress fiber formation and focal adhesions at the edges were observed when single epithelial cells were cultured on fibronectin patterned substrates in Δ, V, T, and Y shapes, where the degree of non-adhesive space underlying the cells influenced adhesive signatures and actomyosin bundling (Théry et al., 2006). Micro-islands of large size (10,000 μm^2^) or small size (1,024 μm^2^) have been shown to trigger osteoblastic or adipogenic differentiation in MSC by modulating endogenous RhoA activity (McBeath et al., 2004). Similar results were observed when diverging differentiation pattern of MSCs to adipocytes or osteoblasts by modulating cytoskeletal tension when cultured on a patterned shapes of the same area (Figure 1B) with differences in subcellular curvature (e.g., pentagon and flower) or aspect ratio (e.g., squares and rectangles), while transcript analysis revealed a role for c-Jun N-terminal Kinase (JNK), Extracellular related Kinase (ERK1/2) and elevated WNT signaling in the differentiating cells (Kilian et al., 2010; Lee et al., 2013). ES or iPS cell colonies are usually comprised of a heterogenous cell population, which is collectively pluripotent, but are composed of mixtures of differentially primed cells. These random mouse ES cell populations were reported to spatially organize themselves in a defined manner when cultured under circular or elliptical geometric confinement highlighting a role for geometry in regulating the spatio-temporal activity of the cells (Blin et al., 2018). Geometry influenced the underlying signaling pathways through WNT, Nodal and BMP signaling (Chhabra et al., 2018), providing a clear example of how paracrine and autocrine signals feed into pathways associated with biophysical characteristics of the microenvironment. Along with spatial regionalization of the previously heterogenous cell populations, various sizes of the patterns were also reported to improve the differentiation efficiency of iPS cells toward a particular cell type: Endothelial cells (Kusuma et al., 2017), neural cells (Xue et al., 2018), or cardiomyocytes (Myers et al., 2013; Ma et al., 2015) during biochemical induction. These reports provide evidence of how mechanotransduction influences multiple cell and tissue level processes as a function of geometry (Figure 1B), stressing the importance of synthetic model platforms to investigate embryonic mechanobiology underlying development.

### From Micro to Nano: Complementarity of Nanoscale Topography

In addition to the role of mechanics and geometry in coordinating tissue form and function, the topography underlying cells will influence adhesive and protrusive features during morphogenesis. Native tissue cells are presented with nanoscale topographical features by their ECM in conjunction with mechanics and geometry influencing their adhesive and migratory behaviors. The effect of anisotropic surface features has been studied on various kinds of cells and their altered behavior in culture by a phenomenon termed as “contact guidance” which affects the cell's alignment and polarization. Cell specific alignment, elongation, actin rearrangement and neuronal lineage specification was observed in polarized cells like neural stem cells when they are cultured on nanogratings (Figure 1B) (Rajnicek et al., 1997; Yang et al., 2013). Variations in cytokine production, morphology, and migration has been observed in fibroblasts, endothelial, and smooth muscle cells (Andersson et al., 2003; Bettinger et al., 2009). MSCs cultured on raised islands or nanopits (Figure 1B) exhibited differentiation toward osteoblastic and fibroblastic cell types and caused increase in fibrillar adhesion length (Dalby et al., 2007). These studies demonstrate the importance of interactions between integrin receptors and nanotopographical features to direct adhesion related signaling, and diverse functional outcomes. Similar alignment and differentiation patterns were observed to alter ES cell behavior without the help of cytokines or growth factors (Lee et al., 2010). While mES cells were demonstrated to maintain their self-renewal abilities on a nanopatterned PDMS substrate (Jeon et al., 2012), human ES cells cultured on nanostructured glass surface were reported to induce a regulatory signal which influences various cell behaviors including self-renewal (Chen et al., 2012). In this study, glass wafer surfaces were prepared with various shapes and roughness levels using photolithography and reactive ion etching (RIE). The ES cells exhibited branched morphology on the smooth surfaces and a more compact morphology and adhesion selectivity on different levels of roughness in the nanorough surfaces in comparison (Chen et al., 2012). Abagnale et al. highlighted the role of nanotopography in iPS cell colony morphology and orientation, and the underlying pathways which were responsible for their self-organized patterning caused by cytoskeletal localization. They seeded human iPS cells on groove-ridge structures and reported (i) organization and elongation of the colonies, (ii) varied expression of pluripotency markers, and (iii) distinct YAP and TAZ localization in the cytoskeletal region during BMP4 mediated differentiation (Abagnale et al., 2017). However, for a complete understanding of spatial migratory behavior of pluripotent cells as a function of topography, new model systems that control nanotopography in 3D are emerging as promising platforms (Culver et al., 2012).

These findings demonstrate how topographical cues can guide cell differentiation and alter their functions at the cell-material interface. Various parameters like cell and tissue size and shape (Werner et al., 2017), cell density (Kempf et al., 2016), soluble factors (Ding et al., 2003), and stiffness (Choi et al., 2016) play a cumulative role toward tissue modeling *in-vitro* (Figure 1B). However, the ability to deconstruct their individual effects and coupling the outcomes together to reconstruct the *in-vivo* events is required to explore a complex process like human embryonic development.

## Micropatterning Assemblies of Pluripotent Stem Cells for Models of Development

Experiments performed by direct manipulation of an extracted mouse embryo, like that done by Hiramatsu et al., reveal insightful information into the developmental stages (Hiramatsu et al., 2013; Arora et al., 2016; Nowotschin and Hadjantonakis, 2018). However, obvious challenges present themselves while working with mouse embryos—small litter size, lesser experimental opportunities and physiological complications of working with live animals. The challenges around live human embryological research are more complex, and have an ethical and legal aspect to them (Pera, 2017). Due to these limitations, alternative methods to recapitulate key developmental milestones during embryogenesis *in-vitro* have been in the pipeline. These models provide a reproducible platform for high quality imaging and quantification opportunities. Most of these models deploy embryonic stem cells to provide a high-throughput platform to elucidate the effects of mechano-geometric cues utilized to direct cell differentiation in the microenvironment. These include 2-dimensional models—which harness the self-organizing properties of embryonic stem cells (Warmflash et al., 2014), in conjunction with biophysical properties of the microenvironment (Deglincerti et al., 2016b; Blin et al., 2018), and 3-dimensional models—which involve the self-assembly of pluripotent cells into embryonic organoids, gastruloid, or blastoids while encapsulated within a 3D biomaterial matrix (Shao et al., 2017; Simunovic and Brivanlou, 2017; Beccari et al., 2018).

The design criteria underlying these cell based models are largely guided by the physical architecture of the mouse embryo which ensures robust restriction of BMP signaling, a key signaling pathway in embryogenesis (Zhang et al., 2018) to orchestrate the spatial organization of ES cells in a gastrulating embryo. Soon after implantation, as the PS is formed in the epiblast, the posterior embryo shows first activation and expression of Brachyury (T/Bra), where the cells break symmetry and initiate EMT toward gastrulation (Sasaki et al., 2016). *In vitro* studies have facilitated the deconstruction of many key events preceding gastrulation including: lineage-specific molecular marker expression, symmetry breaking, emergence of primitive streak (PS), and Epithelial-to-Mesenchymal transition (EMT) (Morgani et al., 2018). Many of these processes can be experimentally controlled through microengineered model systems in the laboratory (Vianello and Lutolf, 2019).

**Figure 2:**
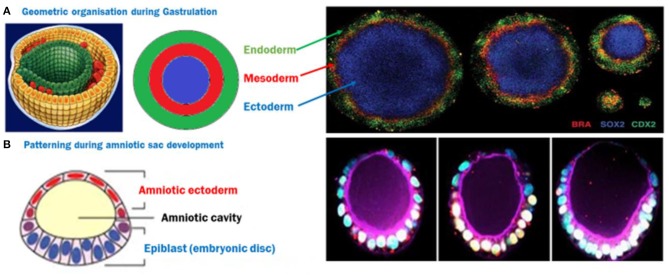
**(A)** ES cells spatially organize themselves into three germ layers following BMP4 induction, depending on the size of the circle, creating a 2D gastrulation model. Image reused with permission (Deglincerti et al., 2016b) Nat Protoc 2016. **(B)** A semi-3D model of post-implantation amniotic sac embryoid (PASE)—The closest human model recapitulating multiple post-implantation embryogenic events. Image reused with permission (Shao et al., 2017) Nat Communications, 2017.

In a 2D system, mouse or human ES cells have been observed to self-organize and pattern Brachyury positive populations in a shape dependent manner (Deglincerti et al., 2016b; Beccari et al., 2018; Blin et al., 2018). Circular micropatterns, coated with a protein to assist cell attachment on an otherwise inert surface allowed ES cells to self-organize themselves after BMP4 induction into concentric populations of cells representing all three germ layers and extra-embryonic like cells, where factors like colony size, geometry/curvature, and cell density were implicated in patterning (Figure 2A; Warmflash et al., 2014). This phenomenon was attributed to an “edge effect” caused by the underlying signaling dynamics of BMP4, Activin/Nodal, and Wnt pathways with corresponding receptor localization (Chhabra et al., 2018; Martyn et al., 2019). These studies introduced a 2D monolayer platform to understand the contribution of the mechanics of the local environment to gastrulation.

This self-organization property of mES cells was also deployed to form more complex embryo-like structures or embryoids where some key events of development were reproduced *in-vitro* (Heemskerk, 2019). Recent work in this direction demonstrated appearance of PS and AP axis, as well as Hox genes activation corresponding with the *in-vivo* sequence of events, by the self-organization of mES cells into quasi- spherical aggregates followed by axis formation and germ-layer specification (Beccari et al., 2018). Induced pluripotent stem cells have gained popularity as an alternative or an addition to such studies, since they could be derived from somatic cells and were proven to have a pluripotent state comparable to embryonic stem cells, allowing them to differentiate into any cell types of the body (Takahashi et al., 2007), with a few dissimilarities reported (Bilic and Belmonte, 2012). Nevertheless, being a cell source which could obviate the limitations of working with ES cells (Siegel, 2004), they have been used in several mammalian developmental models. Moreover, biomaterials have been proven to have the ability to control the microenvironmental architecture and direct iPS cell fates (Abagnale et al., 2017); their use in developmental models has been well-reviewed (Higuchi et al., 2015; Tsou et al., 2016; Ma et al., 2018). The contributions of biomaterials and their tunable physical properties have been deployed to induce morphogenesis and differentiation in hiPS cells to create cardiac tissue models (Myers et al., 2013; Ma et al., 2015; Wang et al., 2018), neuroectodermal tissue models (Xue et al., 2018), skeletal disease modeling (Salazar-Noratto et al., 2016), endothelial cells, and pericytes (Kusuma et al., 2017).

The human iPS cells based developmental model which is closest to an embryo is the PASE—post-implantation amniotic sac embryoid (Shao et al., 2017). In this model, hiPS cells form polarized cysts which differentiate into amniotic ectodermal cells as a function of matrix softness as well as the composition of coated ECM solution (Figure 2B). In some of these cysts, a localized expression of Brachyury has also been reported, indicating a hallmark step during gastrulation (Shao et al., 2017). However, models deploying the tri-lineage differentiation abilities of hiPS cells are quite limited and still largely unable to address some basic questions like the direct effect of architecture and geometry as well as underlying pathways due to the complexities and interdependence of these cues in a 3-dimensional structure resembling an embryo. Recently, a microfluidic device which has parallel channels, with cell loading and induction medium flow, was reported to mimic human epiblast formation and 3D amniotic development using hES and hiPS cells, capitalizing on their self-assembly and organization properties (Zheng et al., 2019). New advances in microfabrication will pave the way to platforms that control multiple microenvironment parameters to emulate a 3D outcome similar to the *in-utero* organization of ES cells, and allow micro analysis of the effect of these cues. Emerging strategies to recreate the uterine constraint have been discussed in a recent review (Vianello and Lutolf, 2019).

## 3D Bioprinting of Pluripotent Stem Cells Towards Developmental Models

Three-dimensional (3D) bioprinting provides an experimental opportunity to rapidly recreate 3D cellular microenvironments which may assist with the intrinsic organization and differentiation behavior of PSCs in a controlled manner. Using various bioprinting techniques, incorporating biochemical factors as well as depositing biomechanical aspects of the desired tissue type, results in 3D multi-cellular structures and composition of the early embryo. These multi-layered constructs have the potential to better represent the 3D complexity of tissue (Moroni et al., 2018). As reviewed by Skeldon et al. (2018), there are a host of new bioprinting techniques being employed in the scientific community for fabricating tissue constructs for regenerative medicine, tissue or disease modeling and drug testing. Recently there have been several advances in 3D printing of tissue-mimetic architectures that carry significant promise for iPSC applications. For instance Miller and colleagues demonstrated a facile method using stereolithography of simple visible-light polymerised hydrogels to fabricate interwoven channels that mimic functional lung tissue (Grigoryan et al., 2019). Feinberg and colleagues developed a new printing approach based on freeform reversible embedding of suspended hydrogels (FRESH) where they were able to print functional heart chambers with collagen-based inks into baths of gelatin colloids that could be dissolved away after fabrication (Lee et al., 2019). Extending this approach into iPSC applications, Skylar-Scott et al. (2019) demonstrated printing of vascular channels within a bath of iPSC derived organoids. In this way, the organoids serve as the colloids allowing high fidelity printing within a matrix of tissue. In addition to these reports, multiple groups have used stem cells or stem cell-derived progenitor cells for various tissue or organoid development models using 3D bioprinting (Ong et al., 2018; Silva et al., 2019).

In the context of embryonic tri-lineage differentiation, the use of hPSCs for 3D printing is still in its infancy. Human PSCs will differentiate into all cell types of the body, a property which makes them ideal for coaxing fate determination in a defined microenvironment. However, single-cell dissociation, which is a prerequisite in most 3D bioprinting methodologies, induces apoptotic signals and reduces survival rates of both hES cells and hiPS cells, as both require cell-cell contact in a tight cluster to survive. However, this issue has been countered by the usage of Rho-kinase (ROCK) inhibitor along with highly specialized PSC media and reagents (Ohgushi and Sasai, 2011). Pioneering work in this area performed by Faulkner-Jones et al., where they used simple inkjet live cell printing technology to create controlled sizes of 3D hES spheroids (Faulkner-Jones et al., 2013) and then went on to create hepatocyte-like cells to produce mini 3D livers using both hES and hiPS cells (Faulkner-Jones et al., 2015). Alginate micro encapsulation also emerged as a 3D platform to differentiate ES cells into pancreatic cells (Richardson et al., 2014) where the 3D encapsulation and induced differentiation of naive ES cells was 20 times more efficient as compared to 2D cultures. The same technique has also been optimized to promote EB formation and directed neuronal differentiation when hES were encapsulated after a treatment with ROCK inhibitor, suggesting a three-dimensional setting promotes neural (ectodermal) lineage (Sidhu et al., 2012). The field has made significant progress in directing lineage specific outcomes, including successful differentiation of iPSCs toward hepatocyte like cells creating a hepatic model (Ma et al., 2016; Pettinato et al., 2016), cartilage engineering (Nguyen et al., 2017) and fundamental studies tracking the effects of biomaterials in guiding differentiation patterns of iPS cells using laser bioprinting (Koch et al., 2018) or extrusion based printing (Gu et al., 2017; Abelseth et al., 2019).

As embryonic lineage commitment patterns have been a major focus for developmental biology, this was soon followed by creation of uniform and pluripotent embryoid bodies (EB) from mouse ES cells using extrusion based 3D bioprinting technology (Ouyang et al., 2015). 3D EB bodies specifically are believed to be a useful model to study the specifics of multilineage differentiation in humans, and hence been tried and tested using various methods using ES cells (Dias et al., 2014). However, developmental models in 2D and 3D are still largely based on the self-organizing mouse and human ES cells, and working toward an iPS cell-based model study using 3D bioprinting for a development or gastrulation model poses considerable challenges.

## Conclusion and Outlook

The role of physical forces in embryonic development has been studied in model systems for fish (Petridou et al., 2019), amphibians (Shawky et al., 2018), and avians (Saadaoui et al., 2018) providing evidence for the irrefutable contributions of mechanics in the development process. In the case of mammals and their *in-utero* development, the embryo is believed to be more susceptible to local physical forces, geometry and general biomechanics because of their embedded development. Some recent publications have provided evidence of the evolutionarily conserved role of mechanics-linked dependence of expression of genes involved in major milestones of mammalian development (Pukhlyakova et al., 2018), effects of stresses imparted by embryonic confinement on the establishment of body axis (Hiramatsu et al., 2013) and highlighted the effects of geometric patterning of embryonic cells and linked these effects to the appearance of early signaling gradients (Warmflash et al., 2014; Blin et al., 2018; Zhang et al., 2018). In an ideal model system, the self-organizing ability of hPSCs would be deployed in parallel to high resolution 3D bioprinted architectures, where biomechanical properties and biochemical composition provide spatiaotemporal cues that mimic the signaling during embryogenesis (Martin et al., 2019). Nevertheless, there are considerable advantages to simple 2D and pseuodo-3D models that enable the researcher to experimentally dissect individual biophysical and biochemical parameters and evaluate their contribution toward the differentiation and patterning behavior of hiPS cells. A model with tunable physical properties would be useful to analyse whether cumulatively these cues could induce the intrinsic self-organization and differentiation behavior of iPSCs without chemical induction and to study underlying paracrine signaling. Moreover, the use of engineered hydrogel biomaterials more closely emulates physiological conditions of the extracellular matrix, with tunable parameters such as stiffness and topology. The ease of handling and reproducibility using these systems allow a high number of experimental opportunities, toward deconstructing the matrix structure and cell function relationships underlying development.

## Author Contributions

PS and KK researched the literature, wrote the article, and assembled the figures.

### Conflict of Interest

The authors declare that the research was conducted in the absence of any commercial or financial relationships that could be construed as a potential conflict of interest.
